# Systemic Immunosuppression for Limbal Allograft and Allogenic Limbal Epithelial Cell Transplantation

**Published:** 2019-12-01

**Authors:** Juan Carlos Serna-Ojeda, Sayan Basu, Jayesh Vazirani, Yonathan Garfias, Virender S. Sangwan

**Affiliations:** 1 Instituto Visión Láser, Aguascalientes, Mexico.; 2 Tej Kohli Cornea Institute, L. V. Prasad Eye Institute, Hyderabad, Telangana, India.; 3 Center for Excellence in Cornea and Ocular Surface Disorders, Excel Eye Care, Ahmedabad, India; 4 Instituto de Oftalmología Conde de Valenciana, Mexico City, Mexico; 5 Dr. Shroff’s Charity Eye Hospital, Daryaganj, New Delhi, India

**Keywords:** Limbal Stem Cell Deficiency, Limbal Allograft, Limbal Epithelial Cell, Immunosuppressive Therapy

## Abstract

Bilateral limbal stem cell deficiency (LSCD) treatment requires the need to obtain allogenic limbal tissue for transplantation. Outcomes of different surgical techniques depend on multiple factors, including the underlying etiology, ocular surface, eyelid status and used surgical intervention. Some of the management options for bilateral LSCD include cadaveric, living related or living non-related conjunctival limbal allograft (CLAL), keratolimbal allograft (KLAL), allogenic cultured limbal epithelial transplantation (CLET) and allogenic simple limbal epithelial transplantation (SLET). Systemic immunosuppressive therapy plays a pivotal role in survival of transplanted tissue. The present review focuses on different systemic immunosuppression protocols for limbal allograft and allogenic limbal epithelial cell transplantation, with specific emphasis on different surgical techniques and their outcomes. We included all reports with details of different systemic immunosuppression protocols for limbal allograft and allogenic limbal epithelial cell transplantation. Oral cyclosporine A at different doses is the most commonly used immunosuppressive agent in limbal allograft and allogenic limbal epithelial cell transplantation. However, different studies using oral mycophenolate mofetil and tacrolimus also reported good results. In conclusion, systemic immunosuppression protocols for limbal allograft and allogenic limbal epithelial cell transplantation are not standardized. Further studies regarding different surgical techniques should assess outcomes and adverse effects of such protocols.

## INTRODUCTION

Limbal stem cells located at limbal epithelial crypts within the limbal palisades of Vogt, play a fundamental role in maintenance of the corneal epithelium through proliferation and migration of new cells [1]. Several diseases can cause limbal stem cell deficiency (LSCD), including chemical injuries, Steven-Johnson syndrome (SJS), vernal keratoconjunctivitis (VKC), ocular cicatricial pemphigoid (OCP), contact lens use, ocular surface tumors, congenital aniridia, etc. [2]. Clinical findings in LSCD include conjunctivalization of the cornea, vascularization, chronic and persistent inflammation, irregular epithelial surface and recurring erosions with persistent epithelial defects and ulceration [3].

In comparison with unilateral LSCD, bilateral LSCD offers a bigger challenge for treatment, as there is no healthy limbus in either eye [4]. This creates the need to obtain allogenic limbal tissue for transplantation. Some of the management options for bilateral LSCD include cadaveric, living related or living non-related conjunctival limbal allograft (CLAL), keratolimbal allograft (KLAL), allogenic cultured limbal epithelial transplantation (CLET) and allogenic simple limbal epithelial transplantation (SLET) [4-6]. 

Outcomes of these techniques depend on multiple factors, including the underlying etiology of LSCD, ocular surface, eyelid status and used surgical intervention [7, 8]. However, systemic immunosuppressive therapy plays a pivotal role in survival of transplanted tissue. Transplantation of an allogenic limbal graft to a densely-vascularized area containing a lot of Langerhans cells increases the risk of rejection [9, 10]. Different studies have used and optimized immunosuppressive protocols according to their experiences. 

The present review focused on different systemic immunosuppression protocols for limbal allograft and allogenic limbal epithelial cell transplantation, with specific emphasis on different surgical techniques and their reported outcomes.

## METHODS

This was a review performed by searching the PubMed database in June 2017 using the following search words; stem cell transplantation, limbal stem cells, stem cell deficiency, ocular surface reconstruction, limbal allograft and allogenic limbal epithelial cell transplantation. The search was restricted to publications in English or publications with English abstracts from 1990 to 2017. Relevant articles found in the reference lists were also included. We included all reports with details of different systemic immunosuppression protocols for limbal allograft and allogenic limbal epithelial cell transplantation.

## RESULTS

**Conjunctival Limbal Allograft**

In both living-related and living-nonrelated CLAL, free grafts including limbal tissue are harvested from the healthy eye and transplanted to the diseased eye [11]. Being one of the first techniques to treat LSCD, immunosuppressive management for patients undergoing this procedure has also been evolved. 

Initial reports of CLAL for eyes with bilateral surface disorders without systemic immunosuppression reported 25% rejection rates, specifically for incompatible or non-available data regarding HLA donor-recipient pairs. Kwitko found that patients with favorable course were either HLA identical or haplo-identical (50% identity) with their donors, thereby proposing HLA-matched allogeneic transplantation as a promising method [12]. Another report declaring long-term results of HLA-matched living related-CLAL in 39 eyes with bilateral ocular surface disease, showed that at one-year follow-up, visual acuity improved in 46.2%, with 48.7% achieving an ambulatory vision and in 84.6% stabilization of the corneal surface occurred [13]. However, other authors evaluated the use of an initial high dose of intravenous methylprednisolone and oral prednisolone with systemic cyclosporine A administered at doses between 1.5 and 5 mg/kg/day and subsequent tapering to a maintenance dose for patients receiving transplantation of limbal tissue from a HLA-matched relative donor [14]. Results showed that 80% of patients had improvement of corneal epithelium and reduction of vascularization, suggesting that low-maintenance dose of cyclosporine is helpful in living related-CLAL [14]. 

Oral cyclosporine has been used in different studies, with an initial dose of 3 to 5 mg/kg, sometimes targeting blood levels to 100 to 150 ng/mL, and tapering to 2 to 4 mg/kg [15-17]. However, a high failure rate achieved with this therapy in severe chemical burns and SJS for both compatible (80%) and non-compatible living related-CLAL (92.3%) [15, 16]. Underlying etiology in these patients was definitely important, with patients presenting with LSCD secondary to chemical burn or mustard gas keratopathy, having less failure rates; whilst for SJS patients, satisfactory ocular surface reconstruction was reported in only 20% of cases, attributed to a high rate of postoperative local complications like infection [15, 17, 18]. Thus, failure of transplant may be more related with the underlying indication, rather than just the immunosuppression protocol. On the other hand, positive results were reported in a pediatric patient with SJS with severe cicatricial disease and a totally keratinized ocular surface, with the use of systemic cyclosporine and living related-CLAL [19]. Also, higher doses up to 10 mg/kg of body weight in divided doses, with addition of diltiazem 90 mg as an adjunct to increase serum levels of cyclosporine have been used for the treatment before visual rehabilitation with penetrating keratoplasty [20]. 

Different systemic protocols appeared, like the use of tacrolimus 4 mg twice daily and mycophenolate mofetil 1 g twice daily by tapering after 6 months for a patient with LSCD due to sclerokeratitis treated with living related-CLAL, showing improvement in visual acuity and a stable ocular surface on follow-up [21]. 

**Keratolimbal Allograft**

KLAL involves transfer of conjunctival and limbal tissue from a cadaver donor eye to an eye with severe LSCD. This technique has become one of the most accepted operations to regenerate the ocular surface. Although transplantation of limbal tissue from living related donors with HLA-matched recipients was attempted to avoid systemic immunosuppression, eventual recurrence of vascularization on long-term follow-up in the initially successful cases led to the adaptation of immunosuppressive protocols [22]. Use of adequate systemic medication is important, as results of allograft transplantation are worse than autografts in terms of both corneal epithelialization and corneal clarity [23]. 

Oral cyclosporine A has also been used as systemic immunosuppressive therapy in most early studies regarding KLAL. This agent was used for unique or co-adjuvant therapy, or in response to a rejection episode [24]. Case-series using cyclosporine A in loading doses and then gradually tapering reported restoration of a stable ocular surface and healing in 65% to 75% of patients with diverse LSCD etiologies [25, 26]. 

Different dose regimens have been tested for systemic cyclosporine. Holland reported a high-dose protocol for patients with diverse etiologies of LSCD undergoing KLAL, using 5 to 7 mg/kg per day [27]. Another protocol used was starting with 5 to 10 mg/kg before surgery and then maintaining serum levels of 100 to 150 ng/mL for one month, decreasing the doses to less than 5 mg/kg if serum level was above 150 ng/mL, or when kidney or liver function test results were out of normal range. For patients having good acceptance for cyclosporine A, the serum levels were maintained between 30 and 100 ng/mL for more than six months [28, 29]. Evaluation in patients with SJS, OCP, chemical and thermal burns reported that 51% had corneal epithelialization and 60% improved two or more lines of visual acuity, having better results in patients with burns [29]. A study that used this initial high-dose immunosuppression for at least 6 months, and tapering after that until 1 year, reported only nausea and vomiting in few cases attributed to cyclosporine [30]. 

Lower doses of oral cyclosporine A, at a dosage of 3 to 5 mg/kg per body weight starting before or at the time of operation, and then tapering to 1 to 2 mg/kg in long-term follow-up, demonstrated an overall graft survival rate of 54.4% at 1 year, 33.3% at 2 years and 27.3% at 3 years in one report, and a survival of ambulatory vision of 53.6% at 3 years and 44.6% at 5 years in another study [31, 32]. However, the two groups reached different conclusions, with the first one finding no difference in KLAL survival between patients treated or not treated with long-term cyclosporine A. This is probably because they administered oral immunosuppression in the first years after treatment only to high-risk patients and after occurrence of acute allograft rejection [31]. The other group achieved a low survival rate and mycophenolate mofetil was added [32]. A study in a large cohort of KLAL patients evaluating clinical characteristics of immunologic reactions, found rejection of the limbal graft in 13% of patients using intravenous cyclosporine A 3 mg/kg starting one day before surgery and continuing for 1 week, followed by oral medication 5 mg/kg and maintained for several months [33]. They suggested reducing the initial dose to 2 mg/kg in patients aged 70 years or older [34]. 

A similar protocol was implemented in another study of KLAL with simultaneous PK by Shi et al. in 2008. They administered oral cyclosporine A 3 to 4 mg/kg daily for 6 months and tapered to 2 to 3 mg/kg daily for at least 1 year. This study showed limbal stem cell rejection at one year in 39% (9/23 patients), and final survival of 87%, compared with 74% KLAL survival from other trials evaluating simultaneous procedures [32, 35]. However, corneal graft survival rates differed in the two studies, reported to be 56.5% at a mean follow-up of 32 months, and 13.7% at 36 months, respectively [32, 35]. This was probably due to differences in surgical indications and exclusion criteria. With similar doses of systemic cyclosporine, Shimazaki and coworkers found 35.5% of endothelial rejection in patients with simultaneous KLAL and PK [36]. 

Other immunosuppressive drugs reported in limbal transplants include tacrolimus FK506, as a more potent immunosuppressant analog to cyclosporine A. Short-term success with use of tacrolimus in KLAL in six patients with LSCD has been reported [37]. Azathioprine 100 mg/day in addition to cyclosporine for patients with SJS or aniridic keratopathy has also been used [38, 39]. 

The Cincinnati Eye Institute and University of Cincinnati described a systemic immunosuppression protocol addressing limbal transplantation with an approach similar to solid organ transplantation. They used high-dose oral corticosteroids, in addition to oral tacrolimus initiated at 4 mg twice daily tapered after 6 months and oral mycophenolate mofetil 1 g twice daily tapered after 12 months, depending on the results of the ocular surface [10]. Patients were also followed by a renal transplantation team. They added valganciclovir for prophylaxis against cytomegalovirus and trimethoprim/sulfamethoxazole for pneumocystis carinii [10].

This same immunosuppressive therapy was used in different studies to treat patients with LSCD secondary to soft contact lens wear and to methamphetamine production–related ocular injuries, with a high rate of symptom resolution, improvement in best-corrected visual acuity (BCVA) and ocular surface stabilization in the first group, but a more challenging prognosis in the second because of severe inflammation, accompanying eye comorbidities and psychological treatment [40, 41].

In Cincinnati study, the incidence of rejection was 31.1% in a mean follow-up of 62.7 months, which was lower to other studies using only oral cyclosporine, compared to adding systemic tacrolimus and mycophenolate mofetil [42]. They also compared their results with a smaller series of 12 eyes by Liang and coworkers, finding rejection in only 16.7% over a mean follow-up of 61.2 months. In this study, patients received 1 gram of oral mycophenolate mofetil and 1 mg of tacrolimus two times a day starting before surgery. The doses were adjusted according to response of the ocular surface and systemic adverse effects. The doses were tapered to 0.5 gram (mycophenolate mofetil) and 0.25 mg/kg (tacrolimus) per day and discontinued in case of graft failure or adverse effects [43]. 

A group from Iran used cyclosporine A 5 mg/kg daily, then decreased to 3 mg/kg daily after some weeks and discontinued after 1.5 to 2 years, with mycophenolate mofetil 1 g two times a day started after surgery and continued for minimum 6 months in a group of patients undergoing KLAL in mustard gas-induced LSCD [44, 45]. The authors reported an acute rejection in 10% (4 of 40 eyes), resulting in only 4% graft failure (1 of 40 eyes), but with 3 eyes requiring a repeated KLAL because of a recurrence of primary disease for a mean follow-up of 19.6 months [45]. Also, when combining KLAL before or concomitantly with lamellar keratoplasty, KLAL survival was about 84.4% with improvement in corneal surface in a mean follow-up of 41.4 months [44].

The Cincinnati Procedure assessed living related CLAL and KLAL in eyes with severe bilateral ocular surface failure and conjunctival deficiency [46]. They evaluated 24 with the most common etiology of SJS who were followed for more than a year. They reported stabilization in the ocular surface in 54.2%, improving in 33.3%, and failing in 12.5% of eyes, with almost 80% of patients requiring a subsequent keratoplasty. [46] Later, the same group reported the Modified Cincinnati Procedure, which is a combination of conjunctival limbal autografts (CLAU) and KLAL for unilateral severe ocular surface disease, in a case series from patients with chemical burns, reporting that 73% had BCVA of 20/80 or better, and ocular surface stability in 82% [47].

Oral mycophenolate mofetil 1 gram twice a day has also been indicated only in cases of acute rejection or adverse events of cyclosporine A [48]. Other immunosuppressive medications have been used. A case report described the use of intravenous immunoglobulin in a patient with antibody-mediated KLAL rejection resistant to systemic and topical corticosteroids, with an improvement in clinical signs and symptoms [49].

An important consideration in combination of different systemic medications is the possibility of adverse events. These include alterations in blood tests such as anemia, hyperglycemia, elevated creatinine and elevated liver function tests, which are usually normalized by decrease in doses or discontinuation of the medication [50]. In their 10-year experience with this protocol, the group from Cincinnati reported severe adverse events (cardiovascular events) in only 1.5% of patients, and minor adverse events (transient biochemical abnormalities, increased cardiovascular risk, among others) in 14% of patients [10]. Also, even with the combination of medications, acute and chronic rejections remain an important issue among the complications on KLAL, and they may appear even as a late presentation [51, 52]. 

**Allogenic Cultured Limbal Epithelial Transplantation**

Evolution of tissue engineering for reconstruction of the ocular surface in bilateral LSCD allowed creation of an allogeneic corneal epithelial stem cell sheet. However, most allogeneic corneal epithelial transplantation usually fails without immunosuppressive therapy [53]. This usually occurs because allogeneic cultivated limbal cells can be target of immunological reactions, even if there are no antigen-presenting cells in the cultivated epithelial sheets [54]. Although a meta-analysis for ex vivo CLET showed no difference in success rate and visual acuity compared with autograft, immunosuppression is critical to the success of allogeneic grafts [55]. 

Systemic cyclosporine A is also a common therapy in allogeneic CLET, but dosage and duration of treatment are not well-known [56]. In a case-series of ex-vivo expanded cultured corneal epithelial stem cells on a modified amniotic membrane surface, Schwab reported a successful outcome defined as restoration or improvement of vision, along with maintenance of corneal surface stability in 4 allogenic transplants treated with cyclosporine A 200 mg daily for immunosuppression, in a follow-up of 6 to 19 months [57, 58]. However, cyclosporine A was early decreased or discontinued in all the patients, in one after 3 weeks due to a bacterial keratitis, in another after a subsequent ocular surgery 1 month later, and in 2 due to renal complications [58]. Therefore, subsequent studies attempted to reduce the doses of systemic immunosuppression. Daya et al. used 3 mg/kg tapered to 2 mg/kg after 2 weeks, and then maintained indefinitely in a mean follow-up of 28 months for ex vivo expanded stem cell allografts, showing overall improvement in 70% of patients, and visual acuity improvement in 40% [59]. Shimazaki and coworkers used the same initial dose of 3 mg/kg systemic cyclosporine A, continued for minimum of 6 months, with systemic levels maintained at 100 to 150 ng/mL, or blood cyclosporine levels of 800 to 1000 ng/mL after 2 hours [60-63]. In a study of thirteen eyes with severe limbal deficiency due to SJS, OCP and chemical burns, the epithelium regenerated and covered the ocular surface in 61.5% of eyes, but only 46.2% maintained the same result at last follow-up visits [60]. In an extended case series, they reported a poorer outcome, with most patients (7 of 12) in the allogenic group failing to achieve corneal epithelization [61].

With similar cyclosporine doses of 2 to 5 mg/kg/day, but maintained for 6 months and then tapered until it was stopped after the first year, Prabhasawat and coworkers found a high rate of success (85.7%, 6/7 eyes) with stable epithelialization in allograft cases, more commonly with LSCD due to chemical burns [64]. More recently, Shortt et al. assessed a daily dose of oral cyclosporine A 3.5 mg/kg continued for 6 months in patients with LSCD, showing improvement in the clinical parameters and visual acuity, restoration of a normal corneal phenotype, and appearance of normal confocal microscopy in 71% of allografts [65]. 

Zakaria and coworkers, prescribed oral cyclosporine A 2 x 125 mg/day for a similar duration of time, tapered with later discontinuation at one-year after transplant in 3 allogenic transplant recipients, and two achieved reversion to a persistent intact epithelium and a functional improvement [66].

Some studies added systemic cyclophosphamide due to its fast-acting effect, to prevent postoperative inflammation and rejection. Koizumi and coworkers used cyclosporine (150 mg/day) and cyclophosphamide (100 mg/day) in addition to oral corticosteroid, stopping the last two 1 to 2 months after surgery, but continuing a low-dose cyclosporine (25–50 mg/day) for 6 months [67, 68]. With cultivated corneal epithelial transplantation to treat patients with severe LSCD of diverse etiologies, including acute and chronic SJS, acute and chronic chemical injuries, OCP and drug-induced pseudopemphigoid, the authors reported improvement in visual acuity in all eyes after surgery. In this study, 10 of 13 eyes improved two or more lines 6 months after the operation and 3 patients developed epithelial rejection during the follow-up period [67]. 

The same group using autologous serum derived cultivated corneal epithelial transplantation for the treatment of severe ocular surface disease of diverse etiologies, used lower doses of immunosuppressive drugs, with oral cyclophosphamide (50 mg/day) and cyclosporine (100 mg/day), discontinuing both medications between 1 and 3 months after surgery, and reported that during the follow-up period, the corneal surface of all patients remained stable [69]. With this protocol, those authors confirmed later that the allogeneic transplanted cells survived longer on the corneal surface maintaining ocular surface integrity, with the evaluation of two excised grafts of patients, 42 and 75 months, respectively, after the initial graft transplantation [70]. 

In the series of patients with allogeneic transplantation of cultivated limbal epithelium treated by Pauklin and coworkers, they also used 360 to 720 mg mycophenolate mofetil twice per day for 18 months in one patient because the original immunosuppressive therapy was not tolerated, unlike other patients who were treated with cyclosporine A 100 mg twice per day for 12 to 15 months [71]. They reported a significant increase in visual acuity, and a stable central corneal surface restoration in 71.4% of eyes treated by allogeneic transplantation [71].

**Allogenic Simple Limbal Epithelial Transplantation**

SLET is a recently reported technique in which a 2x2 mm strip of donor limbal tissue is obtained from the healthy eye and divided into eight to ten small pieces to distribute them over an amniotic membrane placed on the cornea of the recipient eye [72]. Long-term results in the treatment for unilateral cases of chemical burns have shown promising outcomes; however, no studies have evaluated its application to bilateral cases, were an allogenic limbal tissue is taken and harvested in the recipient cornea and using immunosuppressive therapy to increase the survival of limbal cells [73]. 

A case report about a patient with bilateral total LSCD after a chemical injury and treated with allogenic SLET and postoperative management of topical and oral corticosteroids, reported an episode of acute rejection, managed with a single pulse dose of 500 mg intravenous methylprednisolone and hourly topical prednisolone acetate 1% eye drops, and then continued with tapering doses of oral prednisolone without additional immunosuppressive drugs [74]. A study evaluated the results of allogenic SLET without additional systemic immunosuppression in patients with acute severe chemical injuries, to achieve rapid epithelialization and prevent adverse effects of delayed epithelial healing; these patients achieved faster epithelialization, and only 5% required a subsequent lamellar keratoplasty [75]. Further studies in allogenic SLET may also address the results of systemic immunosuppression protocols.

## CONCLUSION

Evaluating different systemic immunosuppression protocols for limbal allograft and allogenic limbal epithelial cell transplantation is difficult due to a wide diversity of variables including number of patients, etiology of LSCD, surgical technique, previous or concomitant ocular surgeries, follow-up time and outcome definitions. Table 1 compares the outcomes of some of the published large studies (>35 eyes) that include systemic immunosuppression regimen and their outcomes.

Oral cyclosporine A at different regimens is the most commonly used immunosuppressive agent in limbal allograft and allogenic limbal epithelial cell transplantation. However, different studies using oral mycophenolate mofetil and tacrolimus also reported good results. Further studies of different surgical techniques should assess outcomes and adverse effects of such protocols. 

**Table 1:** Published Large (>35 eyes) Studies of Limbal Allograft and Allogenic Limbal Epithelial Cell Transplantation With Systemic Immunosuppression Regimen.

**Author**		**Year**	**Surgical Technique**	**Systemic immunosuppression protocols **	**Details, causes of LSCD**	**Eyes; n**	**Mean follow-up**	**Outcomes**
**Tsubota et al [** **29** **]**	1999		Ring-shaped limbal tissue with epithelial stem cells, multiple surgeries in some cases)	Oral cyclosporine A 5 to 10 mg/kg of body weight and tapered.	Severe ocular-surface disorders, Stevens-Johnson syndrome and ocular pemphigoid most common.	43	Mean of 1163 days	51 % had corneal epithelialization, of which 35% with clear cornea.
**Solomon et al ** **[** **32** **]**	2002		KLAL+ AMT, in 58.9% simultaneous PKP.	Oral cyclosporine A 5 mg/kg of body weight and tapered.	Retrospective study of Patients with LSCD, most common cause was chemical burns.	39	Mean follow-up was 34.0 months (range, 12 to 117.6)	Survival of KLAL: 76.9% at 1 year, 47.4% at 3 years, and 23.7% at 5 years. Survival of PKP: 47.8% at 1 year.
**Maruyama-Hosoi et al ** **[** **33** **]**	2006		KLAL	Intravenous cyclosporine A 3 mg/kg starting 1 day before surgery and continuing for 1 week, followed by oral cyclosporine 5 mg/kg.	Retrospective study to analyze characteristics of immunologic reactions. Stevens-Johnson syndrome and ocular cicatricial pemphigoid were the most common ones.	85	Average follow-up was 46.6 months.	55.3% had clear grafts at last examination.
**Javadi et al [** **45** **]**	2011		lr-CLAL and KLAL	Oral cyclosporine A 5 mg/kg of body weight daily and then reduced to 3 mg/kg daily after 6 months.	Retrospective study of patients with delayed-onset mustard gas keratitis with simultaneous or subsequent corneal transplantation.	72	Mean follow-up after lr-CLAL was 65.6 months (range, 30 to 102) and 19.6 months (range, 13 to 61) after KLAL.	25% in the lr-CLAL required KLAL. The rejection-free graft survival rate was 52.2% in lr-CLAL and 80.7% in KLAL at month 10.
**Holland et al [** **10** **]**	2012		Most common KLAL (83.8%)	Most common tacrolimus, mycophenolate mofetil, and prednisone (75%)	Retrospective study of patients undergoing ocular surface stem cell transplantation	225	Mean 53.9 months (range, 3.6 to 147.3 months).	77.2% had a stable ocular surface.
**Ang et al [** **42** **]**	2013		Most common KLAL (80.6%)	Oral prednisone, tacrolimus and mycophenolate mofetil	Retrospective study to analyze ocular surface stem cell transplantation rejection. Aniridia most common indication.	222	Mean follow-up of 62.7 months (range, 12.0 to 158.3 month)	Rejection occurred in 31.1% eyes; severe rejection in 19.4% and low-grade rejection in 11.7%.
**Baradaran-Rafii et al [** **51** **]**	2013		KLAL	1 g of oral mycophenolate mofetil and 4 mg of tacrolimus twice a day starting 1 week preoperatively and tapered after 12 months.	Retrospective review of complications of KLAL in different etiologies of LSCD, more common chemical injuries.	45	Mean follow-up time was 26.1 months (range, 6 to 48 months).	At last follow-up, 73.4% had a stable ocular surface.
**Parihar et al [** **30** **]**	2017		Allogenic ex vivo cultivated LSCT (25 eyes) vs KLAL (25 eyes)	Oral cyclosporine A 5 to 10 mg/kg of body weight and tapered.	Chemical / thermal burns injuries most common etiology in both groups	50	1 year of follow-up	Absence of conjunctival revascularization (LSCT: 60%; KLAL: 54.54%) comparable in both groups.

Abbreviations: AMT: Amniotic membrane transplantation. KLAL: keratolimbal allograft. lr-CLAL: living-related conjunctival limbal allografts. LSCD: Limbal stem cell deficiency. LSCT: Limbal stem cell transplantation. PKP: Penetrating keratoplasty; n: number; %: percentage; mg/kg: milligrams per kilogram of body weight; vs: versus; g: gram; mg: milligrams.
